# Nemabiome metabarcoding shows a high prevalence of *Haemonchus contortus* and predominance of *Camelostrongylus mentulatus* in alpaca herds in the northern UK

**DOI:** 10.1007/s00436-024-08226-w

**Published:** 2024-05-03

**Authors:** Osama Zahid, Meghan Butler, Andy Hopker, Emily Freeman, Livio M. Costa Júnior, Umer Chaudhry, Neil Sargison

**Affiliations:** 1 Royal (Dick) School of Veterinary Studies and Roslin Institute, Easter Bush Veterinary Centre, University of Edinburgh, Midlothian, EH25 9RG UK; 2 Pathology Department, Federal University of Maranhão, São Luís, Maranhão Brazil; 3 School of Veterinary Medicine, St. George’s University, St. George’s Grenada, West Indies Grenada; 4 Department of Comparative Biomedical Sciences, School of Veterinary Medicine, University of Surrey, Guildford, GU2 7AL UK; 5 Current Address: Galedin Veterinary, Kelso, TD5 7BH UK

**Keywords:** Alpaca gastrointestinal nematodes, Nemabiome metabarcoding sequencing, *Haemonchus contortus*, *Camelostrongylus mentulatus*, Internal transcribed spacer 2

## Abstract

Gastrointestinal nematodes (GINs) are a common threat faced by pastoral livestock. Since their major introduction to the UK in the early 1990s, South American camelids have been cograzed with sheep, horses, and other livestock, allowing exposure to a range of GIN species. However, there have been no molecular-based studies to investigate the GIN populations present in these camelids. In the current study, we sampled nine alpaca herds from northern England and southern Scotland and used high-throughput metabarcoded sequencing to describe their GIN species composition. A total of 71 amplicon sequence variants (ASVs) were identified representing eight known GIN species. *Haemonchus contortus* was the most prevalent species found in almost all herds in significant proportions. The identification of *H. contortus* in other livestock species is unusual in the northern UK, implying that alpacas may be suitable hosts and potential reservoirs for infection in other hosts. In addition, the camelid-adapted GIN species *Camelostrongylus mentulatus* was identified predominantly in herds with higher faecal egg counts. These findings highlight the value of applying advanced molecular methods, such as nemabiome metabarcoding to describe the dynamics of gastrointestinal nematode infections in novel situations. The results provide a strong base for further studies involving cograzing animals to confirm the potential role of alpacas in transmitting GIN species between hosts.

## Introduction

Gastrointestinal nematodes (GINs) impact animal health and welfare through both direct pathological and indirect immune-mediated effects (Stromberg and Gasbarre 2006), leading to a reduction in productivity and significant economic losses to the livestock industry worldwide (Charlier et al. 2009; Mavrot et al. 2015). In addition, they substantially increase the industry’s carbon footprint due to consequential higher maintenance requirements for natural resources relative to production output. Mathematical modelling suggests controlling the spread and severity of nematode infections can help to reduce these effects (Kenyon et al. 2013; Nieuwhof and Bishop 2005).

GINs live in complex communities of multiple coinfecting species (Agneessens et al. 1997; Burgess et al. 2012; Giudici et al. 1999; Stromberg et al. 2015; Vlassoff 1976), with each having potentially different epidemiology, pathogenicity, clinical presentation, and drug resistance status (Besier et al. 2016; Whitlock et al. 1980). The composition of these communities is affected by various factors such as temperature and humidity (O’Connor et al. 2006), different farming practices, and the age and immune status of the host animals (Redman et al. 2019). GIN species are adapted to characteristics of different hosts, and many are considered host-specific (Van Wyk et al. 2004), albeit cross-infections of these species can occur. For example, *Haemonchus contortus* is generally considered a small ruminant-adapted nematode with a host preference for sheep but has also been reported in cattle, buffalo, and bison (Avramenko et al. 2018; Ali et al. 2019). Thus, coinfections arising from cograzing domestic and wild animal populations must be considered when exploring GIN infections within specific host populations.

Traditionally, the morphological identification of coprocultured third-stage larvae (L_3_) has been the primary method employed to determine the species composition of nematode populations. This relies on the microscopic examination of the shape, size, and arrangement of various anatomical features in L_3_s (Saidi et al. 2020; Van Wyk et al. 2004; Van Wyk and Mayhew 2013). This classical approach has served as the basis for nematode identification for decades, providing valuable insights into the taxonomy and epidemiology of parasites of farmed small and large ruminants for which keys have been developed. However, this approach is time-consuming and highly dependent on the expertise of skilled taxonomists. In addition, distinguishing between L_3_ of closely related species with overlapping morphological traits poses significant challenges in discerning between species accurately; thus necessitating the exploration of advanced molecular techniques (Roeber and Kahn 2014).

High-throughput metabarcoding of ribosomal nematode DNA provides an example of how next-generation sequencing technology has revolutionised the field of nematode identification. This technique referred to as ‘nemabiome metabarcoding’ involves extracting nematode DNA from nematode eggs, first (L_1_), or third-stage larvae (L_3_) and targeting conserved rDNA ITS-2 primer binding regions to amplify and sequence nematode clade V-specific DNA (Avramenko et al. 2017). The resulting amplicon sequence variants are then filtered and compared to reference databases to identify the nematode species present.

In recent years, nemabiome sequencing has been successfully employed in various livestock hosts including small ruminants (Redman et al. 2019), large ruminants (Avramenko et al. 2017), and horses (Sargison et al. 2022) to determine the composition and diversity of nematode communities. These studies have demonstrated the utility of nemabiome metabarcoding sequencing in accurately identifying gastrointestinal nematode species and providing a comprehensive understanding of the parasitic landscape within these hosts.

Compared to traditional methods, nemabiome metabarcoding sequencing offers several distinct advantages. Firstly, it provides a rapid and high-throughput approach for nematode identification, allowing for the simultaneous detection and characterisation of multiple nematode species within a single sample. This capability is particularly valuable when dealing with mixed infections, or when studying the dynamics of nematode populations over time. Secondly, nemabiome sequencing enhances the accuracy and objectivity of nematode species identification. Unlike traditional morphological methods, which are prone to subjectivity and intra- and inter-observer variability, nemabiome sequencing relies on DNA sequence data, which provides a robust and reproducible basis for species determination. This objectivity minimises biases and increases the consistency of results, enabling comparisons and interpretations across studies. Furthermore, even when compared to species-specific molecular methods, nemabiome metabarcoding has the potential advantage of uncovering amplicon sequence variants representing previously unknown or cryptic species and hybrids. Phylogenetic analysis of amplicon sequence variants can detect subtle genetic variations that may indicate the presence of new, or closely related species (Sargison et al. 2022). Such discoveries contribute to the broader understanding of nematode biodiversity and aid in refining taxonomic classifications.

The use of nemabiome metabarcoding outside of traditional farm animal species is comparatively uncommon, partly because of the difficulty in creating reference databases containing sequences for GINs that might be present in these wildlife, or other unusual hosts. Recently, we developed a sequence library for horses that included the sequences for many wildlife species (Sargison et al. 2022). In the current study, we applied this library to study the presence and proportional abundance of nemabiome amplicon sequence variants of GINs present in alpacas in the northern UK. The work aimed to improve understanding of the GIN communities infecting UK camelid herds, as a basis for improved control strategies.s

## Methods

### Sample collection and processing

Faecal samples were collected from nine alpaca farms in the north of England and south of Scotland between July and November 2018. The freshly voided samples were obtained from the ground of communal defecation sites. Precautions were taken to avoid any cross-contamination for the faeces of any cocrazing animals. Available information was gathered on cograzing and GIN management, as shown in Table 1.

**Table 1:** Average trichostrongyle and *Nematodirus* faecal egg count at the beginning and end of a grazing season, with cograzing and GIN management data

Farm ID	*n*	Trichostrongyle counts (epg)		*Nemotodirus* spp. counts (epg)			Other animals cograzed with the alpacas	Typical anthelmintic treatment history
		Range	Mean	Range		Mean		
1	16	0–12	3.9	0–6		0.8	Wild deer	October (moxidectin); May (moxidectin)
2	11	0–155	23.5	0–14		2.5	Sheep; horse	April (moxidectin)
3	12	0–12	2.8	0–3		1.8	None	November (moxidectin); March (doramectin)
4	15	0–9	1.2	0–12		2.8	Horses	May (febendazole)
5	22	0–22	20.2	0–7		4.2	Donkeys	May (doramectin)
6	7	0–30	9.0	0–3		0.9	Wild deer; sheep; cattle; pigs	None
7	4	3–12	9.8	0–30		8.3	Sheep	March (ivermectin)
8	5	0–18	7.8	0–12		3.0	Sheep; horses; pigs	June (moxidectin)
9	12	0–69	8.0	0–6		0.8	Sheep; cattle	June (febendazole); October (febendazole)

The number of alpacas tested (*n*) and the ranges and means of trichostrongyle and *Nematodirus* FECs (eggs per gram) on each farm are given in this table. Animals reported to cograze with the alpacas and anthelmintic treatments for GINs of the alpacas during the previous 12 months are also shown

Then, 1 g of faeces from each sample was used to perform a faecal egg count (FEC) using a saturated salt flotation and the cuvette method with a detection threshold of 1 egg per gram (epg) (Christie and Jackson 1982). Equal amounts of the remaining faecal material (> 10 g) were pooled for each farm and incubated at about 20 °C for 14 days for L_3_ coproculture. L_3_ were isolated by Baermannisation (Großbritannien 1986) and fixed in 70% ethanol. All of the L_3_ obtained from the coprocultures were used to produce DNA lysates.

For DNA extraction, 1000 μl Direct PCR Lysis Reagent (Viagen), 50 μl of proteinase K (Quiagen) solution, and 50 μl of 1 M dithiothreitol (DDT) were added to create a worm lysis solution. Then, 20 μl of this worm lysis solution was added to each sample and incubated at 60 °C for 2 h, followed by 15 min at 85 °C to inactivate the proteinase K (Evans et al. 2021). The lysates were stored at – 20 °C until further use.

### Adapter and barcoded PCRs

Previously published primers and conditions (Avramenko et al. 2015) were used to amplify the rDNA ITS-2 region. PCR products were purified with AMPure XP magnetic beads according to the manufacturer’s guidelines, followed by the second round of PCR amplification to add unique barcode combinations to each sample using the previously described method (Rehman et al. 2020). Finally, the samples were pooled (10 µl PCR product from each sample) and purified using a Qiagen gel extraction and purification kit, followed by further purification through AMPure XP magnetic beads. Then, 20 μl of the pooled sample was submitted to Edinburgh Genomics for Illumina MiSeq, using a 500-cycle paired-end reagent kit (MiSeq Reagent Kits v2, MS-103–2003) at a concentration of 15 nM with the addition of 15% PhiX Control v3 (Illumina, FC-11–2003). Each resequencing step followed Illumina’s standard protocol.

The numbers of L_3_ recovered varied greatly between farms, and the DNA amount could not be equalised between samples; hence, the results are focused on describing the GIN species present on individual farms, rather than direct proportional comparisons.

### GIN species analysis

**Fig. 1:**
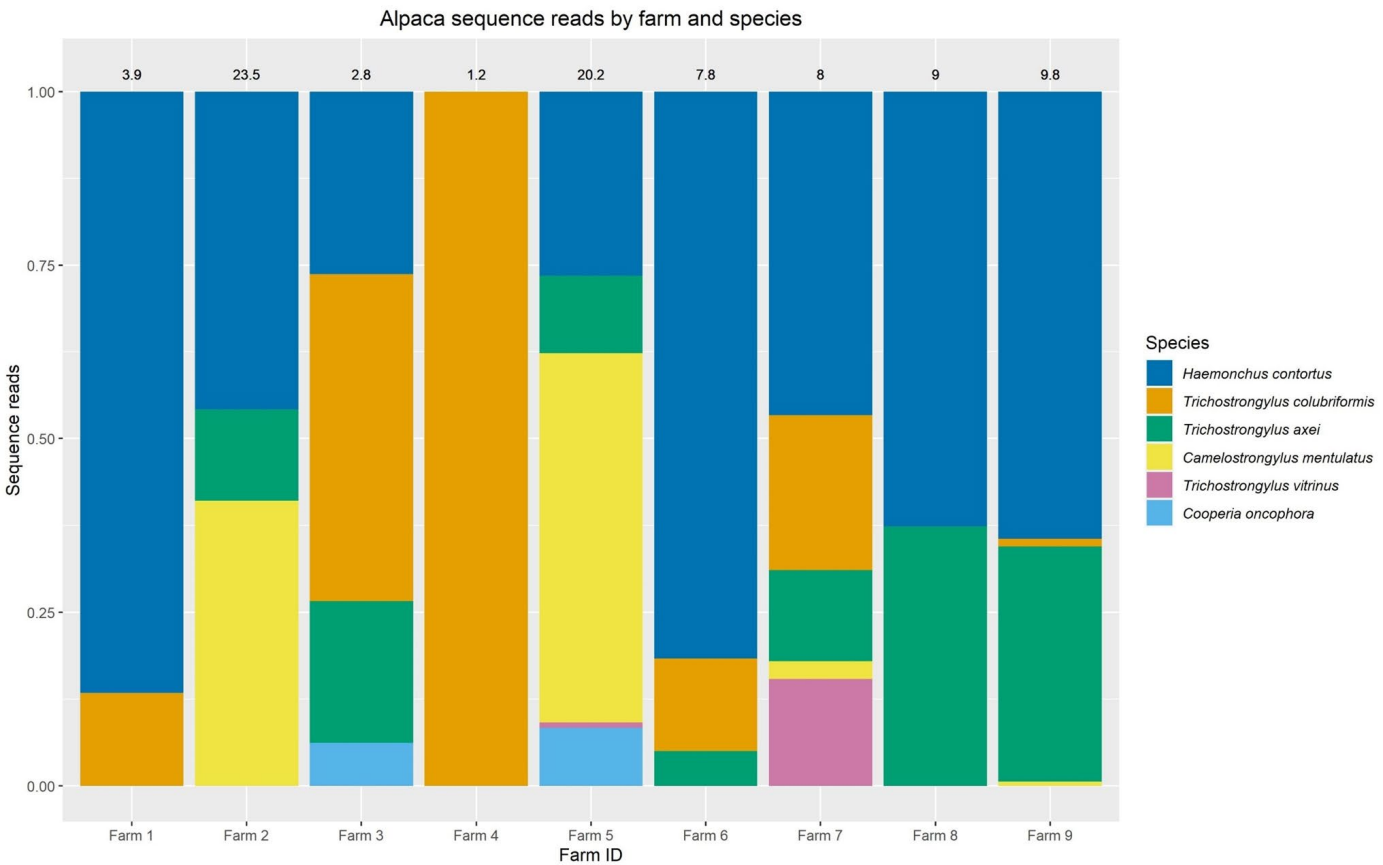
The relative abundance of nematode species on different farms. Each color represents a separate species, and each bar is a different farm. The *x*-axis shows the sequence reads proportion for each GIN species, with different farms on the y-axis. Each farm’s mean faecal worm egg count is shown on the top of each bar. The legend shows the colour of each GIN species as well as their arrangement within the bar chart

**Fig. 2:**
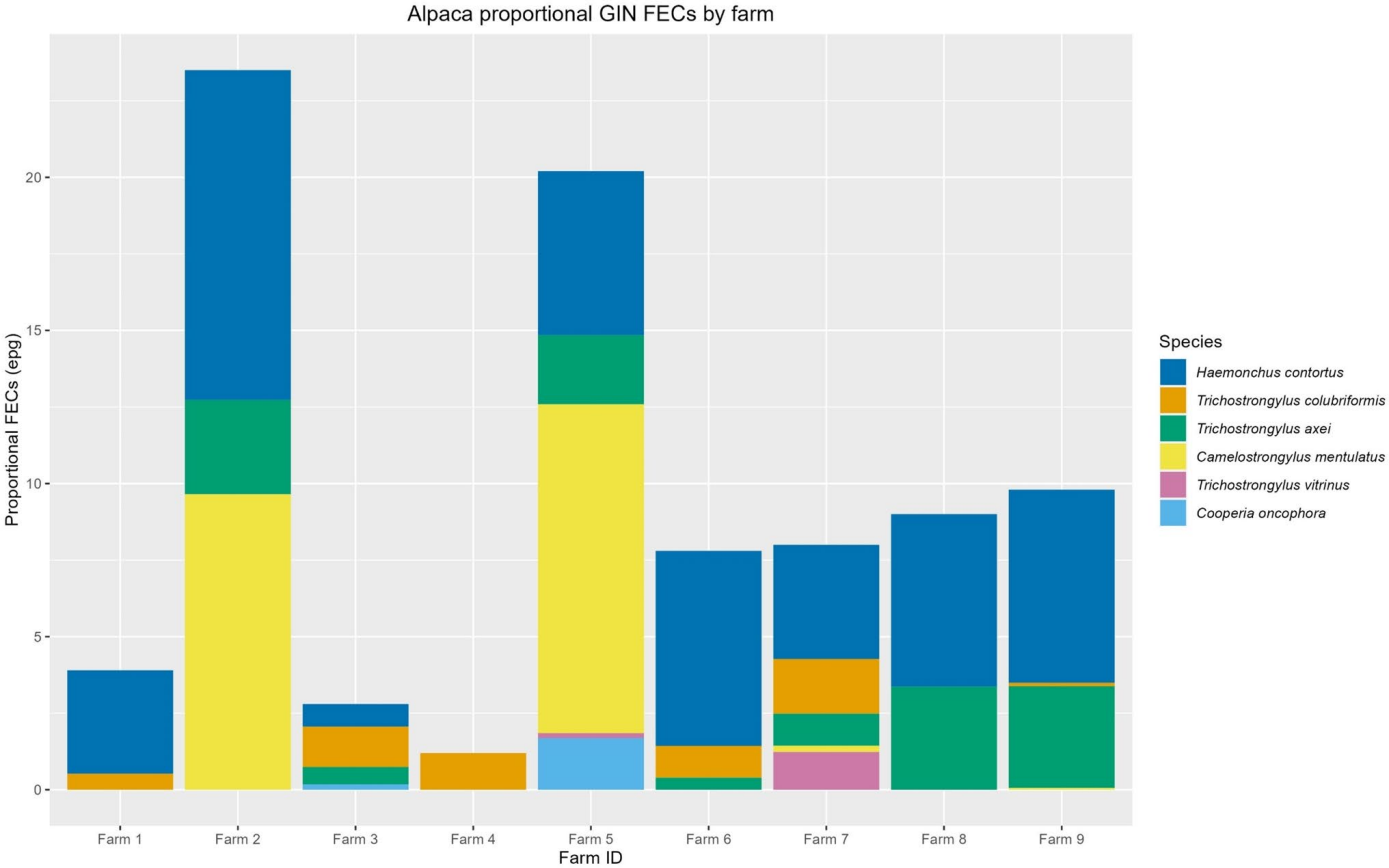
The proportional faecal egg count (FEC) of each nematode species in different farms. Each color represents a separate species, and each bar is a different farm. The *x*-axis shows the proportional FEC for each GIN species, obtained by multiplying the FEC with proportional reads. Different farms are on the *y*-axis. The legend shows the colour of each GIN species as well as their arrangement within the bar chart

The FASTQ files obtained from the post-run Illumina MiSeq processing, representing sequences present in each index-recognised sample, were analysed following the adapted Illumina MiSeq protocols for nemabiome in Mothur v1.39.5 (Schloss et al. 2009). The steps involved joining paired forward and reverse reads and screening sequences shorter than 200 bp, longer than 450 bp, or with any ambiguous bases before they were aligned to a bespoke reference sequence library (https://github.com/drosamazahid/uk_alpaca) containing ruminant, horse, camelid, and wild animal nematode species using a Needleman-Wunsch pairwise alignment method, following the described workflow (https://www.nemabiome.ca/mothur_workflow.html). The sequence library had previously been developed and used to study equine GIN populations, for which there was limited a priori knowledge of what species might be present (Sargison et al. 2022). The sequences were then classified into different species/groups according to the taxonomy file of the reference library using the *k*-nearest neighbor algorithm (knn) method. Finally, a summary file showing a total of about 342,000 (average 38,000 per sample; range 6176–74,168) aligned sequences belonging to different species in each sample was created and inputted to R (R Core Team 2021) for further analysis. Before calculating the relative abundance of different species, any species which had less than 1% of the total reads (3420) was removed to avoid stochastic effects arising from low egg counts and to negate the effects of bleeding/index hoping during the sequencing process, leaving 337,000 total reads (average 37,444 per sample; range 6176–70,951). Correction factors (available at https://www.nemabiome.ca/mothur_workflow.html) were not used, as none are available for *Camelostrongylus mentulatus.* Bar charts were produced to show the proportion reads and intensity of infection (obtained by multiplying the proportion reads with FEC) of GIN species on different farms (Figs. 1 and 2).

### Confirmation of species identity

Sequences of each species were separated, and the identical sequences present at least twice were collapsed using FaBox DNA collapser (https://birc.au.dk/~palle/php/fabox/dnacollapser.php) to obtain amplicon sequence variants (ASVs). A total of 61 ASVs were obtained which were then blasted on NCBI to confirm their identities. These matched 100% with the results obtained from our library. The top five blast results for each ASV, along with their percentage identity match with the previous sequences in NCBI GenBank are available at https://github.com/drosamazahid/uk_alpaca.

### Phylogenetic analysis

**Fig. 3:**
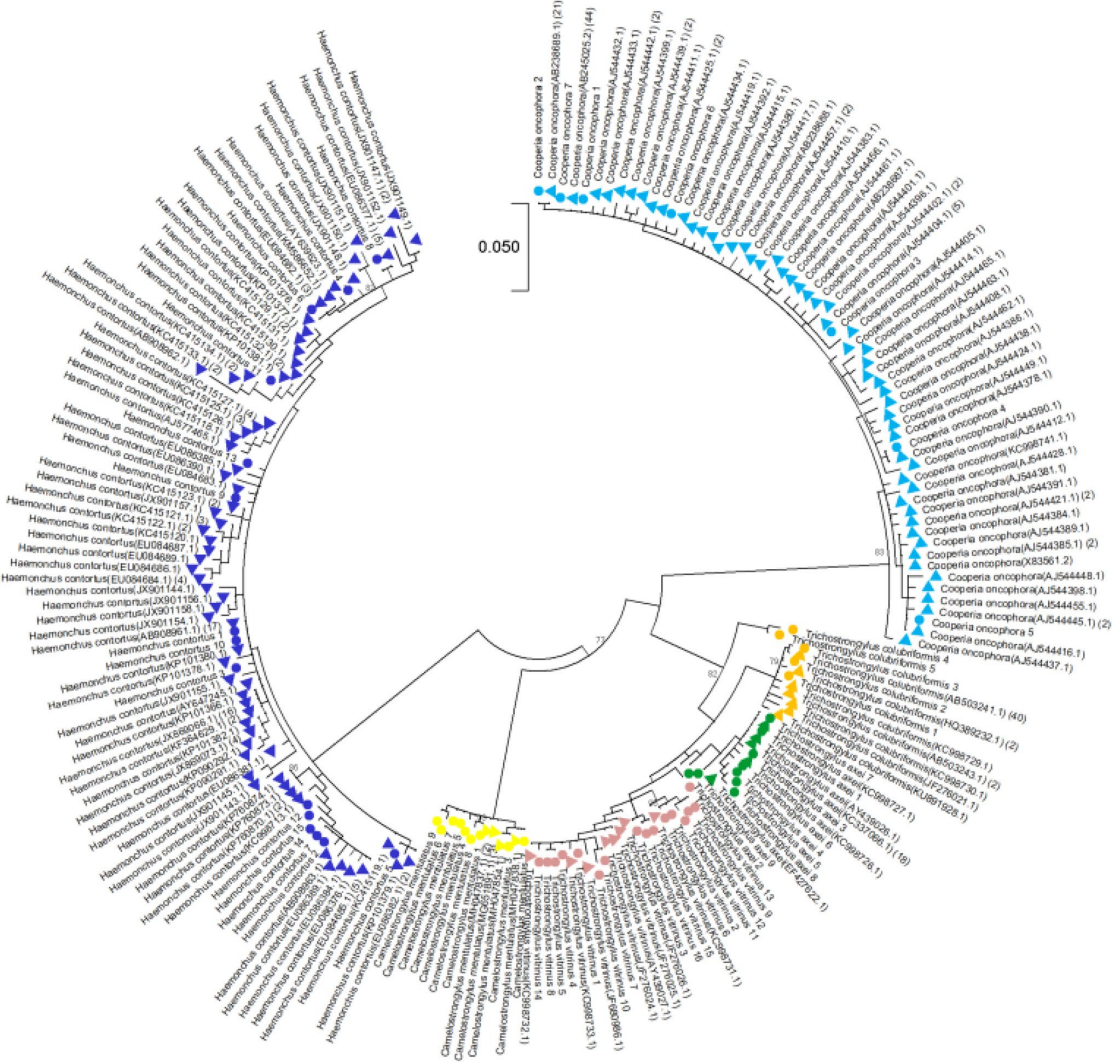
Phylogenetic tree of GIN species found in alpaca herds in the northern UK and the GenBank sequences***.*** The phylogenetic tree of field ASVs and Genbank sequences was constructed using the maximum likelihood method and the Tamura 3-parameter model. The tree with the highest log likelihood (− 25,627.67) is shown. Initial tree(s) for the heuristic search were obtained automatically by applying neighbor-join and BioNJ algorithms to a matrix of pairwise distances estimated using the Tamura 3 parameter model and then selecting the topology with the superior log-likelihood value. A discrete gamma distribution was used to model evolutionary rate differences among sites (5 categories (+ G, parameter = 0.9576)). This analysis involved 207 nucleotide sequences, and there was a total of 362 positions in the final dataset. Evolutionary analyses were conducted in MEGA X. The numbers on branches show bootstrap values. Triangles show GenBank sequences with their accession number identity, while circles represent field ASVs. Each colour represents a different GIN species. The Genbank accession numbers are shown in brackets after the name. The second bracket shows the number of identical GenBank sequences merged to simplify the analysis

Phylogenetic trees of both the field sequences and the NCBI GenBank sequences of all the species found in the field samples were constructed in MEGA X to show where the field sequences aligned with previously reported NCBI Genbank sequences. The GenBank sequences were obtained by manually searching for each species. Partial sequences were removed and duplicates were merged before constructing the tree using the Tamura 3-parameter model (Tamura 1992) The tree with the highest log likelihood (− 25,627.67) is shown in Fig. 3**.** Initial tree(s) for the heuristic search were obtained automatically by applying neighbor-join and BioNJ algorithms to a matrix of pairwise distances estimated using the Tamura 3-parameter model and then selecting the topology with superior log-likelihood value. A discrete gamma distribution was used to model evolutionary rate differences among sites (5 categories (+ G, parameter = 0.9576)). This analysis involved 207 nucleotide sequences, and there was a total of 362 positions in the final dataset.

## Results

### Faecal egg count and management data

The mean FEC results are shown in Table 1. Overall, the counts were low and varied between 1 and 24 eggs per gram (epg) for trichostrongyle eggs and 1 and 8 epg for *Nematodirus* spp. eggs. Cograzing animals and anthelmintic treatments of the alpacas during the previous 12 months are also shown in Table 1. Most of the herds had been treated with one of moxidectin, doramectin, ivermectin and fenbendazole during the previous six months, except farm 6 which had no anthelmintic treatment history. Similarly, all but farm 3 had a history of cograzing with different animals including sheep, horses, donkeys, cattle, pigs, and wild deer. It is worth noting that the study adopted a convenience-based sampling approach; hence, it is possible that the selected farms might not be wholly representative of alpaca herds in the northern UK.

### The abundance of different GIN species

The species analysis confirmed six known GIN species in the nine herds studied (Fig. 1**)**. *Haemonchus contortus* was the most common species, present in all except farm 4, where a very low FEC and consequent L_3_ yield might have accounted for its detection failure. *Trichostrongylus axei* was found in all herds, except those on farms 1 and 4. *Trichostrongylus colubriformis* and *C. mentulatus,* which were present in six (farms 1, 3, 4, 6, 7, and 9) and four (farms 2, 5, 7, and 9) herds, respectively. *Cooperia oncophora* was identified on farms 3 and 5, and *Trichostrongylus vitrinus* on farms 5 and 7.

The GIN populations identified in the herds on farms 5 and 7 showed the greatest diversity with five different GIN species present. Those on farms 3 and 9 were ranked second with four GIN species each, followed by three species each on farms 2 and 6. Only two species each were identified in the herds on farms 1 and 8. Farm 4 showed the least diversity with just one GIN species; albeit the FEC was very low (1.2 epg), and consequently, a minimal number of larvae may have been amplified.

The proportional FEC chart (Fig. 2) shows most species to have similar abundances in different herds, regardless of the total egg count. The most abundant species are *H. contortus* and *T. axei*, while *C. mentulatus* seems to be predominant on farms with relatively higher egg counts.

### Phylogenetic analysis

The ML tree of the field and NCBI Genbank ASVs (Fig. 3) shows the GIN species separated into different clades. The *Trichostrongylus* spp. sit very close to each other in a single large clade. *C. oncophora, C. mentulatus*, and *H. contortus* are separated into their own clades. The tree also shows all field samples sitting with their respective clades of GenBank sequences, supporting the species identity of those ASVs.

## Discussion

Camelids have been kept in increasing numbers in the UK since major imports during the 1990s, especially alpacas which are mostly kept for fibre production, recreational enterprises, and as therapy animals and pets. Nevertheless, except for a few faecal egg counts, copro-cultured larval morphology studies, and post-mortem reports (de B Welchman et al. 2008; Mitchell et al. 2016; Tait et al. 2002), there is little published information regarding their GIN infections and none involving confirmation through molecular techniques.

The current study, based on high-throughput sequencing (Avramenko et al. 2015), provides a robust insight into the GIN species present in UK alpacas. The FECs were low in all the samples, in agreement with the previous studies (Kultscher et al. 2019). This might be partially attributed to the distinct alpaca behaviour of establishing communal defecation and urination sites referred to as latrines (McGregor 2002; McGregor and Brown 2010). Since alpacas do not normally graze around these latrines when sufficient grazing is available, they may avoid acquiring a high level of infection. It is important to note that this is not always the case and that alpacas have been found to have higher FECs (> 300 epg) in some studies (Bedenice et al 2022); which might be attributed to the differences in the location of these latrines. It is not the scope of this study, but it will be interesting to investigate how this behaviour changes the overall composition of GINs in alpacas as it might favour certain GIN species over others. It might also be important to study any cograzing animals, as they may maintain and spread most of the GIN species found, and could affect the challenge and burdens in the camelids.

The low FECs highlight a limitation of the study. Nevertheless, they do not greatly impact our results, because adequate numbers of sequence reads from multiple GIN species were generated from each sample (except for those from farm 4). DNA was extracted from copro-cultured L_3_ to allow easy processing of samples with low FECs. Other methods to isolate DNA directly from faeces (Pafčo et al. 2018), from eggs (Redman et al. 2019), or from L_1_ (Queiroz et al. 2020) have been described along with arguments concerning their ability to represent GIN diversity that is present. However, the choice of method ought not to affect the presence/absence of GIN species, which was the focus of this study. There are correction factors available for some of the GIN species to account for slight differences in the efficiency of DNA amplification (https://www.nemabiome.ca/mothur_workflow.html). However, we did not apply these, primarily because they are unavailable for camelid-adapted GIN species, *C. mentulatus* in particular.

Another potential limitation arises from the methodology of pooling samples for each farm regardless of individual FEC and the lack of DNA quantification before sequencing. While this approach reflects the real-world conditions within farms, it complicates comparisons across different farms. Additionally, the variability in anthelmintic treatment schedules among the farms adds another layer of complexity. For instance, moxidectin was potentially administered within 12 weeks of sampling at farms 1 to 3, which could influence parasite loads and species dynamics. Given these challenges, the study’s results and discussions have been primarily focused on analysing the dynamics of individual GIN species, with particular focus on *H. contortus* and *C. mentulatus*, rather than making broad comparisons between farms.

Most of the GIN species identified in the current study were of presumed small-ruminant origin (*H. contortus* and *Trichostrongylus* sp.), while *C. oncophora* is considered a cattle species. No apparent correlation was seen between the GIN species found and the cograzing or anthelmintic treatment history, albeit the small sample size prevented any kind of statistical analysis.

*Nematodirus lamae* was previously identified in UK Alpacas (Becklund 1963; Mitchell et al. 2016). While *Nematodirus* sp. eggs were identified in the current study, these would not have consistently hatched in the coprocultures (Zajac 2006). Hence, it was not possible to confirm their species identity using molecular methods.

The predominance of *H. contortus* is noteworthy because it is relatively very uncommon in its preferred sheep hosts in the study region in the northern UK (Sargison et al. 2007). Similar results have also been reported in German alpaca herds (Kultscher et al. 2019). The distinct digestive physiology or grazing behaviour of camelids might make them a well-adapted host for *H. contortus*. The investigation of the potential for alpacas to act as a reservoir for *H. contortus* affecting small ruminants could be worthwhile.

*Camelostrongylus mentulatus* was the only camelid-specific GIN species found. Interestingly, it was present on the 3 farms with the highest FECs. This species has previously been reported in UK alpacas (de B Welchman et al. 2008), but its presence has not been confirmed with molecular biology. *Camelostrongylus mentulatus* has also been reported to infect small ruminants in different countries (Beveridge and Ford 1982; de Ybáñez et al. 2003; Hilton et al. 1978; Mayo et al. 2013); hence, it will be interesting to investigate the GINs in sheep and goats cograzing with alpacas in future studies.

In summary, the study demonstrates the value of next-generation resequencing methods to study the composition and diversity of GIN communities in a novel host species. The results confirm the presence of at least six different GIN species in alpacas in the northern UK. The unexpectedly high prevalence of *H. contortus* and predominance of *C. mentulatus* in herds with the highest FECs prompt the need to revise the sustainable GIN control practices for alpacas and cograzing livestock species. The study also shows the importance of molecular techniques such as nemabiome sequencing to describe changing GIN coinfections in different hosts and inform effective and sustainable parasite control.

## Data Availability

The sequence library used, along with the resultant sequences obtained can be accessed on GitHub: https://github.com/drosamazahid/uk_alpaca.

## References

[CR1] Agneessens J, Dorny P, Hollanders W, Claerebout E, Vercruysse J (1997). Epidemiological observations on gastrointestinal nematode infections in grazing cow-calf pairs in Belgium. Vet Parasitol.

[CR2] Ali Q, Rashid I, Shabbir MZ, Shahzad K, Ashraf K, Sargison ND, Chaudhry U (2019). Emergence and the spread of the F200Y benzimidazole resistance mutation in *Haemonchus*
*contortus* and *Haemonchus*
*placei* from buffalo and cattle. Vet Parasitol.

[CR3] Avramenko RW, Redman EM, Lewis R, Yazwinski TA, Wasmuth JD, Gilleard JS (2015). Exploring the gastrointestinal “nemabiome”: deep amplicon sequencing to quantify the species composition of parasitic nematode communities. PLoS One.

[CR4] Avramenko RW, Redman EM, Lewis R, Bichuette MA, Palmeira BM, Yazwinski TA, Gilleard JS (2017). The use of nemabiome metabarcoding to explore gastro-intestinal nematode species diversity and anthelmintic treatment effectiveness in beef calves. Int J Parasitol.

[CR5] Avramenko RW, Bras A, Redman EM, Woodbury MR, Wagner B, Shury T, Liccioli S, Windeyer MC, Gilleard JS (2018). High species diversity of trichostrongyle parasite communities within and between Western Canadian commercial and conservation bison herds revealed by nemabiome metabarcoding. Parasit Vectors.

[CR6] Becklund WW (1963) Lamanema chavezi gen. n., sp. n. and Nematodirus lamae sp. n.(Nematoda: Trichostrongylidae) from the alpaca, Lama pacos, and the vicuña, Vicugna vicugna, in Peru. J Parasitol 1023–1027 14084183

[CR7] Bedenice D, Resnick-Sousa J, Bookbinder L, Trautwein V, Creasey HN, Widmer G (2022). The association between fecal microbiota, age and endoparasitism in adult alpacas. PLoS One.

[CR8] Besier R, Kahn L, Sargison N, Van Wyk J (2016). Diagnosis, treatment and management of *Haemonchus*
*contortus* in small ruminants. Adv Parasitol.

[CR9] Beveridge I, Ford G (1982). The trichostrongyloid parasites of sheep in South Australia and their regional distribution. Aust Vet J.

[CR10] Burgess CG, Bartley Y, Redman E, Skuce PJ, Nath M, Whitelaw F, Tait A, Gilleard JS, Jackson F (2012). A survey of the trichostrongylid nematode species present on UK sheep farms and associated anthelmintic control practices. Vet Parasitol.

[CR11] Charlier J, Höglund J, von Samson-Himmelstjerna G, Dorny P, Vercruysse J (2009). Gastrointestinal nematode infections in adult dairy cattle: impact on production, diagnosis and control. Vet Parasitol.

[CR12] Christie M, Jackson F (1982). Specific identification of strongyle eggs in small samples of sheep faeces. Res Vet Sci.

[CR13] de B Welchman D, Parr J, Wood R, Mead A, Starnes A (2008). Alpaca and llama nematodes in Britain. Vet Rec.

[CR14] de Ybáñez MR, Garijo M, Carpintero M, Martínez-Carrasco C, Ortiz J (2003). *Camelostrongylus*
*mentulatus* in domestic goats from the Iberian Peninsula. J Helminthol.

[CR15] Evans M, Chaudhry UN, Costa-Júnior L, Hamer K, Leeson SR, Sargison N (2021). A 4 year observation of gastrointestinal nematode egg counts, nemabiomes and the benzimidazole resistance genotypes of Teladorsagia circumcincta on a Scottish sheep farm. Int J Parasitol.

[CR16] Giudici C, Aumont G, Mahieu M, Saulai M, Cabaret J (1999). Changes in gastro-intestinal helminth species diversity in lambs under mixed grazing on irrigated pastures in the tropics (French West Indies). Vet Res.

[CR17] Großbritannien MoA (1986) Manual of veterinary parasitological laboratory techniques: 160 S.: Ill. HM Stationery Office (Great Britain. Ministry of Agriculture, Fisheries and Food)

[CR18] Hilton R, Barker I, Rickard M (1978). Distribution and pathogenicity during development of *Camelostrongylus*
*mentulatus* in the abomasum of sheep. Vet Parasitol.

[CR19] Kenyon F, Dick JM, Smith RI, Coulter DG, McBean D, Skuce PJ (2013). Reduction in greenhouse gas emissions associated with worm control in lambs. Agriculture.

[CR20] Kultscher L, Hinney B, Schmäschke R, Joachim A, Wittek T (2019). Current anthelmintic treatment is not always effective at controlling strongylid infections in German alpaca herds. Parasit Vectors.

[CR21] Mavrot F, Hertzberg H, Torgerson P (2015). Effect of gastro-intestinal nematode infection on sheep performance: a systematic review and meta-analysis. Parasit Vectors.

[CR22] Mayo E, Ortiz J, Martínez-Carrasco C, Garijo M, Espeso G, Hervías S, Ruiz de Ybáñez M (2013). First description of gastrointestinal nematodes of Barbary sheep (*Ammotragus*
*lervia*): the case of *Camelostrongylus*
*mentulatus* as a paradigm of phylogenic and specific relationship between the parasite and its ancient host. Vet Res Commun.

[CR23] McGregor B (2002). Comparative productivity and grazing behaviour of Huacaya alpacas and Peppin Merino sheep grazed on annual pastures. Small Rumin Res.

[CR24] McGregor B, Brown A (2010). Soil nutrient accumulation in alpaca latrine sites. Small Rumin Res.

[CR25] Mitchell S, Hopkins B, Corfield C (2016). *Nematodirus*
*lamae* identified in an alpaca in the UK. Vet Rec.

[CR26] Nieuwhof GJ, Bishop S (2005). Costs of the major endemic diseases of sheep in Great Britain and the potential benefits of reduction in disease impact. Anim Sci.

[CR27] O’Connor LJ, Walkden-Brown SW, Kahn LP (2006). Ecology of the free-living stages of major trichostrongylid parasites of sheep. Vet Parasitol.

[CR28] Pafčo B, Čížková D, Kreisinger J, Hasegawa H, Vallo P, Shutt K, Todd A, Petrželková KJ, Modrý D (2018). Metabarcoding analysis of strongylid nematode diversity in two sympatric primate species. Sci Rep.

[CR29] Queiroz C, Levy M, Avramenko R, Redman E, Kearns K, Swain L, Silas H, Uehlinger F, Gilleard JS (2020). The use of ITS-2 rDNA nemabiome metabarcoding to enhance anthelmintic resistance diagnosis and surveillance of ovine gastrointestinal nematodes. Int J Parasitol Drugs Drug Resist.

[CR30] R Core Team (2021) R: a language and environment for statistical computing. R Foundation for Statistical Computing, Vienna, Austria. https://www.R-project.org/

[CR31] Redman E, Queiroz C, Bartley DJ, Levy M, Avramenko RW, Gilleard JS (2019). Validation of ITS-2 rDNA nemabiome sequencing for ovine gastrointestinal nematodes and its application to a large scale survey of UK sheep farms. Vet Parasitol.

[CR32] Rehman ZU, Zahid O, Rashid I, Ali Q, Akbar MH, Oneeb M, Shehzad W, Ashraf K, Sargison ND, Chaudhry U (2020). Genetic diversity and multiplicity of infection in Fasciola gigantica isolates of Pakistani livestock. Parasitol Int.

[CR33] Roeber F, Kahn L (2014). The specific diagnosis of gastrointestinal nematode infections in livestock: Larval culture technique, its limitations and alternative DNA-based approaches. Vet Parasitol.

[CR34] Saidi A, Mimouni R, Hamadi F, Oubrou W (2020). Some larval morphological characteristics of Camelostrongylus mentulatus and Nematodirus spathiger. Ukr J Vet Agric Sci.

[CR35] Sargison N, Wilson D, Bartley D, Penny C, Jackson F (2007). Haemonchosis and teladorsagiosis in a Scottish sheep flock putatively associated with the overwintering of hypobiotic fourth stage larvae. Vet Parasitol.

[CR36] Sargison N, Chambers A, Chaudhry U, Júnior LC, Doyle SR, Ehimiyein A, Evans M, Jennings A, Kelly R, Sargison F (2022). Faecal egg counts and nemabiome metabarcoding highlight the genomic complexity of equine cyathostomin communities and provide insight into their dynamics in a Scottish native pony herd. Int J Parasitol.

[CR37] Schloss PD, Westcott SL, Ryabin T, Hall JR, Hartmann M, Hollister EB, Lesniewski RA, Oakley BB, Parks DH, Robinson CJ (2009). Introducing mothur: open-source, platform-independent, community-supported software for describing and comparing microbial communities. Appl Environ Microbiol.

[CR38] Stromberg BE, Gasbarre LC (2006). Gastrointestinal nematode control programs with an emphasis on cattle. Vet Clin: Food Anim Pract.

[CR39] Stromberg BE, Gasbarre LC, Ballweber LR, Dargatz DA, Rodriguez JM, Kopral CA, Zarlenga DS (2015). Prevalence of internal parasites in beef cows in the United States: results of the National Animal Health Monitoring System’s (NAHMS) beef study, 2007–2008. Can J Vet Res.

[CR40] Tait S, Kirwan J, Fair C, Coles G, Stafford K (2002). Parasites and their control in South American camelids in the United Kingdom. Vet Rec.

[CR41] Tamura K (1992). Estimation of the number of nucleotide substitutions when there are strong transition-transversion and G+ C-content biases. Mol Biol Evol.

[CR42] Van Wyk JA, Mayhew E (2013). Morphological identification of parasitic nematode infective larvae of small ruminants and cattle: a practical lab guide. Onderstepoort J Vet Res.

[CR43] Van Wyk J, Cabaret J, Michael L (2004). Morphological identification of nematode larvae of small ruminants and cattle simplified. Vet Parasitol.

[CR44] Vlassoff A (1976). Seasonal incidence of infective trichostrongyle larvae on pasture: the contribution of the ewe and the role of the residual pasture infestation as sources of infection to the lamb. N Z J Exp Agric.

[CR45] Whitlock H, Sangster N, Gunawan M, Porter C, Kelly J (1980). *Trichostrongylus*
*colubriformis* and *Ostertagia*
*sp* resistant to levamisole, morantel tartrate and thiabendazole: isolation into pure strain and anthelmintic titration. Res Vet Sci.

[CR46] Zajac AM (2006). Gastrointestinal nematodes of small ruminants: life cycle, anthelmintics, and diagnosis. Vet Clin: Food Anim Pract.

